# Characteristics of patients with hematologic malignancies without seroconversion post-COVID-19 third vaccine dosing

**DOI:** 10.1093/biomethods/bpad002

**Published:** 2023-02-14

**Authors:** Sigrun Hallmeyer, Michael A Thompson, Veronica Fitzpatrick, Yunqi Liao, Michael P Mullane, Stephen C Medlin, Kenneth Copeland, James L Weese

**Affiliations:** Advocate Aurora Health, 3075 Highland Parkway, Downers Grove, IL 60515, USA; Advocate Aurora Health, 3075 Highland Parkway, Downers Grove, IL 60515, USA; Aurora Cancer Care, Advocate Aurora Health, 750 W Virginia Street, Milwaukee, WI 53204, USA; Advocate Aurora Health, 3075 Highland Parkway, Downers Grove, IL 60515, USA; Advocate Aurora Research Institute, 3075 Highland Parkway, Downers Grove, IL 60515, USA; Advocate Aurora Health, 3075 Highland Parkway, Downers Grove, IL 60515, USA; Advocate Aurora Research Institute, 3075 Highland Parkway, Downers Grove, IL 60515, USA; Advocate Aurora Health, 3075 Highland Parkway, Downers Grove, IL 60515, USA; Aurora Cancer Care, Advocate Aurora Health, 750 W Virginia Street, Milwaukee, WI 53204, USA; Advocate Aurora Health, 3075 Highland Parkway, Downers Grove, IL 60515, USA; Aurora Cancer Care, Advocate Aurora Health, 750 W Virginia Street, Milwaukee, WI 53204, USA; Advocate Aurora Health, 3075 Highland Parkway, Downers Grove, IL 60515, USA; ACL Laboratories, 5400 Pearl St, Rosemont, IL 60018, USA; Advocate Aurora Health, 3075 Highland Parkway, Downers Grove, IL 60515, USA; Aurora Cancer Care, Advocate Aurora Health, 750 W Virginia Street, Milwaukee, WI 53204, USA

**Keywords:** SARS-CoV-2 mRNA vaccine, COVID-19, vaccine, hematologic malignancies

## Abstract

**Objectives:**

The objective of this study is to explore the characteristics of the subset of patients with hematologic malignancies (HMs) who had little to no change in SARS-CoV-2 spike antibody index value levels after a third mRNA vaccine dose (3V) and to compare the cohort of patients who did and did not seroconvert post-3V to get a better understanding of the demographics and potential drivers of serostatus.

**Study design:**

This retrospective cohort study analyzed SARS-CoV-2 spike IgG antibody index values pre and post the 3V data on 625 patients diagnosed with HM across a large Midwestern United States healthcare system between 31 October 2019 and 31 January 2022.

**Methods:**

To assess the association between individual characteristics and seroconversion status, patients were placed into two groups based on IgG antibody status pre and post the 3V dose, (−/+) and (−/−). Odds ratios were used as measures of association for all categorical variables. Logistic regressions were used to measure the association between HM condition and seroconversion.

**Results:**

HM diagnosis was significantly associated with seroconversion status (*P* = 0.0003) with patients non-Hodgkin lymphoma six times the odds of not seroconverting compared with multiple myeloma patients (*P* = 0.0010). Among the participants who were seronegative prior to 3V, 149 (55.6%) seroconverted after the 3V dose and 119 (44.4%) did not.

**Conclusion:**

This study focuses on an important subset of patients with HM who are not seroconverting after the COVID mRNA 3V. This gain in scientific knowledge is needed for clinicians to target and counsel these vulnerable patients.

## Introduction

Patients with hematologic malignancies (HMs) are at greater risk of severe morbidity and mortality caused by COVID-19 [1, 2]. Patients with HMs who are older, with more comorbidities, specific HM conditions, response statuses, and therapies are even more likely to experience adverse health effects caused by COVID-19, including anywhere from 25% to 34% increased risk of death [3, 4]. This is believed to be due the high levels of immunosuppression making the effects of COVID-19 more severe [3]. Among HM conditions, patients with myeloid malignancies seem to be at highest risk for COVID-19 associated morbidity and mortality, but this varies greatly based on treatment type [2–6].

Moreover, patients with HMs show a lower response to the original two-dose COVID-19 mRNA vaccine series when compared with individuals with other immune suppressing conditions leaving them still more vulnerable to COVID-19 associated morbidity and mortality to their non-HM counterparts [7–9]. A recently published meta-analysis put the pooled proportion of patients achieving a serologic response after a second dose of the vaccine around 87% but was sure to acknowledge that patients with HMs are less likely to mount a response when compared with patients with solid organ cancers (63.7% versus 94.9%), likely due to the type of HM and/or current treatments [10, 11]. Another large study, the CAPTURE study, showed that in patients with HMs, neutralizing antibodies, our current indication of vaccine effectiveness, are significantly reduced, and this appears to hold true with Omicron variants [1, 4, 12].

In the summer of 2021, when the “booster dose” was authorized under the United States Food and Drug Administration (FDA) Emergency Use Authorization (EUA), cancer patients were rightly prioritized for this third shot (3V). A third dose of the mRNA vaccine (“3V”) has shown to be particularly beneficial for HM patients given the reduced response to the previous two dose series [13]. With the increased likelihood of seroconversion among individuals who may have been less responsive to the two dose series there is added protection against COVID-19 associated morbidity and mortality with 3V; however, research shows that this may not be true for all HM conditions and/or underlying treatments and responses.

This current study builds off a previous study conducted by the same team that assessed the characteristics of a smaller cohort of HM patients who had received a 3V dose of the mRNA COVID-19 vaccine, however that study did not focus on the subset of patients who did not seroconvert, specifically [9]. The primary objective of this study is to epidemiologically explore the characteristics of the subset of patient who had little to no change in SARS-CoV-2 spike protein index value levels post 3V (−/−) and compare to the cohort of patients who did seroconvert (−/+) to get a better understanding of the drivers of serostatus, which are not directly related to current or past treatments.

## Materials and methods

This retrospective cohort study analyzed patient data on SARS-CoV-2 spike IgG antibody index values pre- and post-3V across a large Midwestern healthcare system, which consists of 28-hospitals and over 500 sites of care. The ADVIA Centaur sCOVG assay was used to provide semi-quantitative (index value) results for the detection of IgG antibodies to the receptor-binding domain (RBD) of the S1 spike antigen of the SARS-CoV-2 virus. The assay received EUA from the FDA and its performance characteristics were validated internally by ACL Laboratories. Assay sensitivity (positive result agreement) was 95.5% and specificity (negative result agreement) was 99.9%. An index value >1.00 is considered positive for SARS-CoV-2 IgG antibodies [14]. Seroconversion was defined by a second dose value going from negative to a third dose positive value, as indicated by the index value cutoff.

### Participants

This study included 625 patients who had been previously diagnosed with HM within the healthcare system between 31 October 2019 and 31 January 2022 and had received the full dose of a COVID-19 mRNA vaccine, as defined by the CDC, two or more weeks prior to 3V [15]. Post-3V index values were obtained at least 21 days after 3V. Patients were excluded from the study if vaccine status was unknown or considered incomplete, or they had received a primary series vaccination that was not an mRNA vaccine manufactured by Moderna and/or Pfizer-BioNTech. Subjects were identified via Epic EMR database by the research analytics team and de-identified to the study team. This study was determined by IRB to be non-human subjects’ research due to the de-identification of data (#2021-214).

### Variables

Data gathered in this study included: age, race, ethnicity, COVID-19 mRNA vaccine type, COVID-19 infection history prior to 3V of COVID-19 vaccine, days between the second and third dose of COVID-19 vaccine, HM diagnosis, up to four SARS-CoV-2 IgG antibodies results between 28 August 2021 and 31 January 2022, and days between the third dose of COVID-19 vaccine and each IgG result. Age values below 90 were collected as continuous and “Age 90 or older” was recoded as 90. Sex included male and female. Race was collapsed into White, Black, Asian/Pacific Islander, and multi-racial (two or more races). Ethnicity included Hispanic/Latino and non-Hispanic/Latino. Unknown values for Race and Ethnicity were removed from the analysis. COVID-19 mRNA vaccine type included Pfizer-BioNTech and Moderna. Days between vaccine doses were collected as continuous. COVID-19 infection history included all EMR-documented positive SARS-CoV-2 PCR test results for COVID-19 infection. IgG index level values “<0.50” and “>100.00” were recoded to 0.50 and 100.00, respectively.

Diagnosis was collapsed into lymphoid leukemia, non-Hodgkin lymphoma (NHL), multiple myeloma and other plasma cell neoplasms, Hodgkin lymphoma, myeloid leukemia, “Other,” and multiple conditions (Table 1).

**Table 1:** HM inclusion groups by ICD-10

HM grouping	Conditions	ICD-10
Lymphoid leukemia	Lymphoid leukemia	C91.X^a^
	Other lymphoid leukemia	C91.Z
NHL	Other specific and unspecified types of NHL	C85.X
	Non-follicular lymphoma	C83.X
	Mature T/NK-cell lymphomas	C84.X
	Malignant immunoproliferative diseases and certain other B-cell lymphomas	C88.X
	Follicular lymphoma	C82.X
	Other specified types of T/NK-cell lymphoma	C86.X
	Other mature T/NK-cell lymphomas	C84.Z
	Mature B-cell leukemia Burkitt-type	C91.A
Multiple myeloma and plasma cell neoplasms	Multiple myeloma and malignant plasma cell neoplasms	C90.X
Hodgkin lymphoma	Hodgkin lymphoma	C81.X
Myeloid leukemia	Myeloid leukemia	C92.X
	Monocytic leukemia	C93.X
	Leukemia of unspecified cell type	C95.X
	Other leukemias of specified cell type	C94.X
	Other monocytic leukemia	C93.Z
Graft-versus-host disease	Graft-versus-host disease	D89.81X
“Other”	Other neoplasms of uncertain behavior of lymphoid, hematopoietic, and related tissue	D47.X
	Other specified disorders of white blood cells	D72.8X
	Other and unspecified diseases of blood and blood-forming organs	D75.8X
	Eosinophilia	D72.1X

a X refers to 1–9.

### Statistical methods

Descriptive statistics are reported as count (percent) for categorical variables and as median (interquartile range [IQR]) for continuous variables. Demographics and baseline variables are also reported by IgG antibody status before/after receiving 3V dose.

To assess the association between each individual characteristics and seroconversion status, patients were placed into two groups based on IgG antibody status pre and post the 3V dose, (−/+) and (−/−). Categorical variables were compared using a Chi-squared test or a Fisher’s exact test, as appropriate, and continuous variables using a Mann–Whitney *U*-test. *P*-value less than 0.05 were used as an indicator of statistical significance. For categorical variables with more than two levels, *P*-values were calculated for both the global and the pairwise comparisons. Odds ratios (OR) are displayed as measures of association for all categorical variables. Logistic regressions were used to measure the association between HM condition and seroconversion. A multivariable logistic regression model was run to assess significant associations fitting seroconversion on cancer diagnosis adjusting for age, sex, and days between dose 2 and dose 3. Myeloid leukemia and Hodgkin lymphoma were eliminated from the model due to small cell size (Table 4). Achieving seroconversion (−/+) was used as the reference outcome. Male, White, non-Hispanic/Latino, receiving Pfizer-BioNTech as the 3V dose, having previous COVID-19 infection, and NHL were used as reference groups for the respective variables for the OR calculation [3]. NHL was chosen as the reference group because it is the most common condition in our dataset. Data management and analysis were performed by the study team using R 4.1.1 (R Core Team, 2021).

**Table 4.** Association between condition and non-seroconversion in patients with HMs

*N* = 264	OR (adjusted)	95% CI	*P*-value
Condition (Reference: NHL)			
Lymphoid leukemia	0.84	(0.43–1.64)	0.6141
Multiple myeloma and plasma cell neoplasms	0.13	(0.03–0.47)	0.0019*
“Other”	0.09	(0.01–0.72)	0.0235*
Multiple conditions	0.57	(0.30–1.06)	0.0752
Sex (Reference: Male)	0.78	(0.46–1.31)	0.3437
Age	1.00	(0.97–1.03)	0.9638
Days between dose 2 and dose 3	1.00	(0.99–1.00)	0.1731
COVID-19 3V type (Reference: Pfizer-BioNTech)	1.01	(0.57–1.78)	0.9759
COVID-19 infection pre-3V dose (Reference: No)	5.63	(0.44–71.75)	0.1835

Logistic regression with non-seroconversion as the outcome and NHL as the reference condition, adjusting for age, sex, days between dose 2 and dose 3, 3V type, pre-3V infection. Excluded cases with “myeloid leukemia” and “Hodgkin lymphoma”.

* Significant at p-value <0.05.

### Data sharing statement

For original data, contact andrew.marek@aah.org.

## Results

A total of 625 patients with HM had two index value results at least 21 days apart pre and post the 3V dose. Overall, 664 participants had two index value results in the total sample, however 14 patients were excluded for not having two index values results at least 21 days apart and an additional 25 patients were excluded for not having the 3V dose between the two index values. Four patients were excluded from the Race category and seven patients were excluded from the Ethnicity category for missing/unknown values. The median (range) age was 71 (64–78) years, 323 (51.7%) were male, 578 (93.1%) were White, and 605 (97.9%) were non-Hispanic/Latino (Table 2). Three hundred and ninety-one (62.6%) received the Pfizer-BioNTech and 234 (37.4%) received Moderna vaccine as the 3V dose. A total of 21 (3.4%) patients had a COVID-19 infection prior to receiving the 3V dose. The median (range) days between second and third doses of vaccine were 199 (177–224). The mean days between 3V dose and index value 2 were 26.3 (SD = 19.3). In the overall sample, the top three conditions were 28% were diagnosed with NHL, 18.2% diagnosed with LL, and 12.2% diagnosed with multiple myeloma (Table 2).

**Table 2.** Patient demographics

	*N* = 625
Age (median, IQR)	71 (64–78)
Sex	
Male	323 (51.7%)
Female	302 (48.3%)
Race^a^ (*N* = 621)	
White	578 (93.1%)
Black	33 (5.3%)
Asian/Pacific Islander	8 (1.3%)
Multi-racial	2 (0.3%)
Ethnicity^a^ (*N* = 618)	
Non-Hispanic/Latino	605 (97.9%)
Hispanic/Latino Origin	13 (2.1%)
COVID-19 3V type	
Pfizer-BioNTech	391 (62.6%)
Moderna	234 (37.4%)
COVID-19 infection pre-3V dose	
Yes	21 (3.4%)
No	604 (96.6%)
Days between dose 2 and dose 3 (median, IQR)	199 (177–224)
HM condition	
NHL	175 (28.0%)
Lymphoid leukemia	114 (18.2%)
Multiple myeloma and plasma cell neoplasms	76 (12.2%)
Myeloid leukemia	28 (4.5%)
Hodgkin lymphoma	16 (2.6%)
“Other”	34 (5.4%)
Multiple conditions	182 (29.1%)

a Unknown values for race and ethnicity were removed, resulting in the reduced number of participants shown for the variables.

### Comparisons by seroconversion status

**Figure 1.**
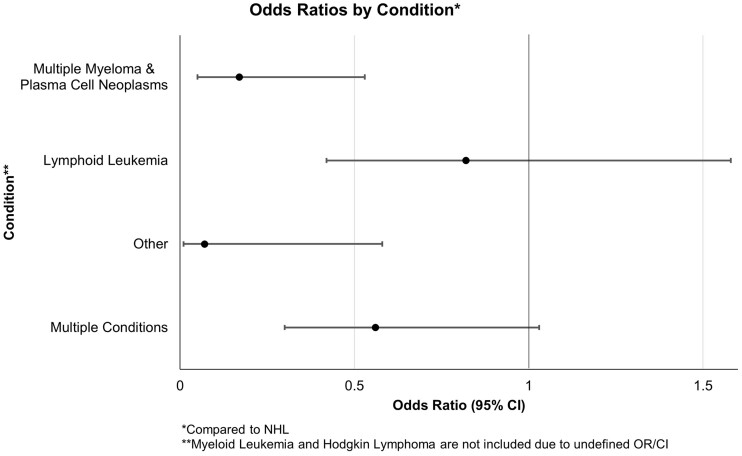
ORs by HM condition.

The following analyses are only comparing the subgroup of HM patients who had a negative serostatus pre-3V, given the lower likelihood of seroconversion when compared with non-HM counterparts. Among the participants who were seronegative prior to 3V, 149 (55.6%) seroconverted after the 3V dose and 119 (44.4%) did not (Table 3). Results show the median days between dose 2 and dose 3 were significantly higher in patients who seroconverted compared with those who did not (*P* = 0.0171). Additionally, HM condition was significantly associated with seroconversion status (*P* = 0.0003). NHL patients have six times the odds of not seroconverting compared with multiple myeloma patients (95% confidence interval [CI]: 1.88–19.12, *P* = 0.0010). NHL patients also have about 14 times the odds of not seroconverting compared with patients diagnosed with the “Other” HM condition group (95% CI: 1.72–112.44, *P* = 0.0021) (Fig. 1).

**Table 3.** Patient characteristics associated with paired IgG results before and after 3V dose

	−/+ (*N* = 149)	−/− (*N* = 119)	ORs (95% CI)^a^	*P*-value
Age (median, IQR)	73 (65–80)	72 (66–77.5)	–	0.7323
Sex				
Male	77 (51.7%)	72 (48.3%)	REF	–
Female	72 (60.5%)	47 (39.5%)	0.70 (0.43–1.14)	0.1485
Race^b^				
White	135 (54.0%)	115 (46.0%)	REF	–
Black	9 (75.0%)	3 (25.0%)	0.39 (0.10–1.48)	0.1532
Asian/Pacific Islander	3 (75.0%)	1 (25.0%)	0.39 (0.04–3.81)	0.6277
Multi-racial	1 (100.0%)	0 (0.0%)	0.00 (0.00, NA)	1.0000
Ethnicity^b^				
Non-Hispanic/Latino	146 (55.5%)	117 (44.5%)	REF	–
Hispanic/Latino Origin	2 (50.0%)	2 (50.0%)	1.25 (0.17–8.99)	1.0000
COVID-19 3V type				
Pfizer-BioNTech	104 (55.3%)	84 (44.7%)	REF	–
Moderna	45 (56.3%)	35 (43.8%)	0.96 (0.57–1.63)	0.8884
COVID-19 infection pre-3V dose				
Yes	1 (20.0%)	4 (80.0%)	REF	–
No	148 (56.3%)	115 (43.7%)	0.19 (0.02–1.76)	0.1746
Days between dose 2 and dose 3 (median, IQR)	199 (182–224)	190 (164–211)	–	0.0171*
HM condition				
NHL	38 (44.2%)	48 (55.8%)	REF	–
Lymphoid leukemia	30 (49.2%)	31 (50.8%)	0.82 (0.42–1.58)	0.5496
Multiple myeloma and plasma cell neoplasms	19 (82.6%)	4 (17.4%)	0.17 (0.05–0.53)*	0.0010*
Myeloid leukemia	0 (0.0%)	1 (100.0%)	–	1.0000
Hodgkin lymphoma	3 (100.0%)	0 (0.0%)	0.00 (0.00, NA)	0.0939
“Other”	11 (91.7%)	1 (8.3%)	0.07 (0.01–0.58)*	0.0021*
Multiple conditions	48 (58.5%)	34 (41.5%)	0.56 (0.30–1.03)	0.0629

a Unknown values for race and ethnicity were removed, resulting in the reduced number of participants shown for the variables.

b Based on Wald test *P*-values for direct differences between the variable level relative to the reference level of the same variable.

* Statistically significant at *P* < 0.05 for Chi-squared test (or Fisher’s exact test) if categorical and Mann–Whitney *U*-test are continuous.

Lastly, to further illustrate the relationship between HM condition and seroconversion, patients with multiple myeloma and plasma cell neoplasms or “Other” HM conditions had lower odds of not seroconverting compared with NHL patients. Put conversely, NHL patients had higher odds of not seroconverting (Table 4).

### Post-3V serostatus

**Figure 2.**
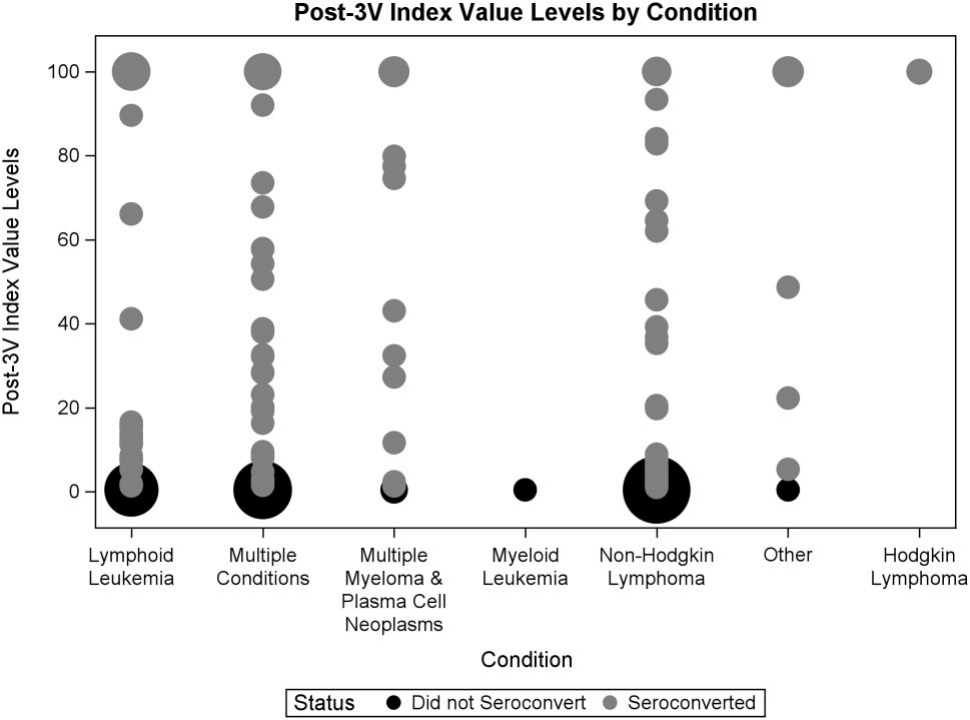
Post-3V seroconversion among HM patients by diagnosis.

Among the cohort of patients who did not seroconvert, 107 (90.0%) patients showed no reaction to 3V as indicated by pre- and post-3V index values. This subgroup had a pre-3V index value and a post-3V index value difference of <0.05 AU/ml. The additional 12 (10.0%) patients showed an average difference in pre- and post-3V of 0.086 AU/ml, indicating almost all the patients who did not seroconvert post-3V had almost no measurable antibody response to 3V using this assay. Among the cohort of patients who did seroconvert, pre- and post-3V average was 43.64, showing a much stronger response to the 3V (Fig. 2). Figure 2 bubbles represent weight/counts.

## Discussion

This study focuses on an important subset of HM patients who did not seroconvert after the COVID mRNA 3V. This study compares the cohort of patients who did not seroconvert (−/−) to the cohort of patients who did seroconvert (−/+) to gain more insight into the differences between these two groups. This study shows preliminary evidence that seroconversion is not driven by age, gender, race, ethnicity, or vaccine type but may be driven by HM condition and days between dose 2 and 3V—with analysis showing seroconversion more likely with patients who had more days between doses 2 and 3 (Table 3). In the multivariate analysis however, only HM condition (multiple myeloma and plasma cell neoplasms and “Other” HM conditions) remained significant, while days between doses are not (Table 4). Only five patients in the pre-3V seronegative group had a confirmed diagnosis of COVID-19 prior to their index value so this analysis was unable to draw any conclusions as to whether previous COVID-19 infection is a driver of seroconversion, due to the limited sample size. Although both cohorts (−/−) and (−/+) had all HM conditions represented, analysis showed that patients with NHL have six times the odds of not seroconverting compared with multiple myeloma patients. Additionally, NHL patients have approximately 14 times the odds of not seroconverting compared with patients diagnosed with the “Other” HM condition group.

This study’s analysis showed that patients who remained seronegative after 3V had little to no reaction, thus raising interesting clinical questions about how to counsel this group of patients (Fig. 2). Given the complexity of immune responses, relying on spike protein alone is likely not enough to assess immunity, especially without looking at T-cell function or neutralizing antibody functional measurements. Current clinical guidance recommends three doses in the primary series and an additional booster, in addition to continued behavioral COVID-19 mitigation strategies [16], especially in immunocompromised populations.

### Limitations

Due to this study data being obtained from a standard of care internal program it lacked T-cell function or neutralizing antibody functional measurements. Additionally, there were only five patients in this sample who had experienced a previous COVID-19 infection so using previous infection as a meaningful variable to assess seroconversion was not possible due to sample size considerations. Further, as part of data derived from a real-world data QI project, the patients were not a homogeneous population with the same treatment schedules, so any influence of patient treatment, medication use, time from diagnosis, or other proxies for disease severity were not included because these data were not collected but such data may complement this analysis and other studies. Lastly, the sample population was predominately white which also may limit generalizability. Future studies should dive more deeply into individual HM patient characteristics as seroconversion may also be a function of disease and/or treatment.

## Conclusion

This study focuses on HM patients who are not seroconverting after the COVID mRNA 3V, suggesting a prioritized population for continued increased behavioral precautions, additional vaccination efforts, including a fifth dose of an mRNA COVID vaccine, as well as passive immunity boosting through monoclonal and/or polyclonal antibodies.

## Ethics statement

Subjects were identified via EMR by the research analytics team and de-identified to the study team. This study was determined by the healthcare system’s IRB to be non-human subjects’ research (non-HSR) due to the de-identification of data (#2021-214).
